# Tertiary lymphoid structures are critical for cancer prognosis and therapeutic response

**DOI:** 10.3389/fimmu.2022.1063711

**Published:** 2023-01-11

**Authors:** Qianqian Zhang, Suhui Wu

**Affiliations:** Department of Obstetrics and Gynecology, Third Hospital of Shanxi Medical University, Shanxi Bethune Hospital, Shanxi Academy of Medical Sciences, Tongji Shanxi Hospital, Taiyuan, China

**Keywords:** tertiary lymphoid structure, cancer, prognosis, immunotherapy, biomarkers, tumor microenvironment

## Abstract

Tertiary lymphoid structures (TLSs) are ectopic lymphocyte aggregates that form at sites of chronic inflammation, including cancers, in non-lymphoid tissues. Although the formation of TLSs is similar to that of secondary lymphoid organs, the pathogenic factors leading to TLS formation in cancerous tissues and the mechanisms underlying the role of these structures in the intra-tumoral adaptive antitumor immune response are not fully understood. The presence of TLSs may impact patient prognosis and treatment outcomes. This review examines the current understanding of TLSs in cancers, including their composition and formation as well as their potential to predict prognosis and therapeutic efficacy. We also summarize strategies to induce TLS formation for cancer treatment.

## 1 Introduction

The clinical outcome of cancer is largely determined by interactions between tumor cells and their microenvironment. In recent decades, it has become increasingly evident that tumors are organs that behave similarly to normal tissues and, in some cases, even outcompete them. According to this viewpoint, tumor biology can only be fully comprehended by studying the specific types of cells within a tumor and the tumor microenvironment (TME) generated during tumor development. The coining of the term “immune contexture”, which is characterized by the type, density, immunological function, and position of tumor-associated immune infiltrates, further emphasizes that the quantity and quality of immune infiltration into the TME can be considered a crucial factor in determining the clinical outcome of cancer patients (1–5).

Lymphoid infiltrating cells, a common component of the TME, have been associated with improved patient survival (6). In particular, the study of lymphocytes with effector and memory functions within primary or secondary tumors and the impact of effector memory CD8^+^ T cell density in tumor lesions on patient prognosis clearly demonstrate the significance of lymphocytes in the TME for tumor control (1, 7). In fact, the majority of immune cells can be found in varying proportions in the center or periphery of tumors (8), with lymphocytes constituting a large proportion of tumor-infiltrating immune cells, although macrophages are typically the most abundant subset (9–11).

Classically, the generation of an effective anticancer immune response involves a series of sequential events comprising a “cancer-immune cycle” (12), which occurs in secondary lymphoid organs (SLOs). This cycle begins with the release of neoantigens during tumorigenesis and their capture by mature dendritic cells (DCs). The DCs then migrate from the tumor lesion to the nearest SLOs to present processed tumor-associated antigens (TAAs) on major histocompatibility complex class I (MHC I) and/or MHC II molecules to CD8^+^ and CD4^+^ T cells, respectively, thereby priming the T cells against neoplasm-specific antigens. Next, primed T cells are translocated to the tumor bed to specifically recognize and bind tumor cells, ultimately ending the fate of their target cancer cells (12). Moreover, activated CD4^+^ T cells interact with B cells receiving the same antigenic stimulus to promote their continued proliferation and differentiation within SLOs. This phenomenon results in the formation of clonally proliferating B-cell populations called germinal centers (GCs), in which B cells undergo a series of developmental processes before migrating to the tumor site to facilitate the killing of tumor cells (13).

Recent studies on the TME have provided a new perspective for understanding antitumor immunity, which can also occur directly at the tumor site in spatially well-organized areas of infiltrative immune cell aggregates called tertiary lymphoid structures (TLSs) (14). TLSs are ectopic lymphoid structures that form in non-lymphoid tissues and are similar in structure and function to SLOs (15). TLSs can be found in the stroma, infiltrative margins, and/or centers of different types of tumors (14, 16–20). The presence of TLSs in tumor lesions can predict clinical prognosis (8, 16, 17) and response to immune checkpoint blockade (ICB) in patients (21, 22), and induction of TLSs in tumors can enhance lymphocyte recruitment and tumor control (23, 24). In contrast, negative outcomes have also been associated with TLSs. For example, TLS-like structures associated with hepatocellular carcinoma (HCC) predict a poor prognosis for patients (25), and a high ratio of CD8^+^ T cells to CD20^+^ B cells is associated with the progression of prostate cancer (26); these findings are indicative of TLS heterogeneity in tumors.

Presently, the clinical significance of TLSs in cancer remains controversial, and the current understanding of the formation and mechanisms of local antitumor immunity is limited. Here, we review the composition, organization, and formation of TLSs in human cancers. By leveraging this information, we describe how TLSs can be used to predict clinical outcomes and immunotherapeutic responses and manipulate anticancer immune responses in a targeted manner.

## 2 Structure and cellular organization of TLSs in cancers

TLSs, also termed tertiary lymphoid organs or ectopic lymphoid structures, are discrete and/or organized aggregates of infiltrating immune cells appearing in non-lymphoid tissues after birth. Unlike primary or secondary lymphoid structures or organs, TLSs do not exist under physiological conditions but seem to be induced at sites of chronic inflammation, for instance, during persistent infections (27), autoimmune diseases (28), transplant rejection (29), and, importantly, cancer (16, 30). The occurrence of TLSs may vary depending on the type of cancer; thus, TLSs are remarkably heterogeneous, and a direct comparison between TLSs in different cancer types is not possible using current datasets, which rely on a range of different molecular markers to recognize TLSs.

The anatomical structure of TLSs is highly plastic and variable, as is their cellular composition, which may comprise a simple population of lymphocytes or a complex, compartmentalized structure resembling lymph follicles in SLOs (15, 16, 27, 31). However, TLSs are not encapsulated by fibrous capsules as SLOs are, and their structure and integrity are maintained by stromal cells (4). Based on specific criteria, well-developed TLSs can contain a substantial diversity of immune cells (only lacking natural killer (NK) cells present in SLOs (32)), which are mainly divided into CD3^+^ T cells and follicular CD20^+^ B cells. Within TLSs, follicular CD20^+^ B cells exist within the inner B-cell zone, which is surrounded by an outer T-cell zone (15, 16, 33) (**Table 1**). The TLS B-cell area resembles lymph node B-cell follicles, with an active GC surrounded by a mantle of naïve B cells interspersed with memory B cells and plasma cells (50). Importantly, the organizational structure and formation of the GC depend on the setting, stage, and site of immune response. In the T-cell compartment, CD4^+^ follicular helper T (T_FH_) cells generally serve as the major subpopulation. T_FH_ cells are present in the vicinity of GC-B cells and may be involved in multiple steps regulating GC development and the differentiation of plasma and memory B cells (51, 52). Other T-cell subsets include CD8^+^ cytotoxic T cells, which are regarded as a vital component of efficient antitumor immunity; CD4^+^ T helper 1 (T_H_1) cells, which can activate other immune cells, such as macrophages, B cells, and CD8^+^ cytotoxic T lymphocytes (CTLs) to induce cytolysis and other effector functions; and FOXP3^+^ regulatory T (T_reg_) cells, which may be correlated with suppressed antitumor immunity and poor patient survival (18, 19, 36, 38).

**Table 1:** Composition of TLSs and markers used for cancer detection.

Structure	Cell types	Molecular markers	Refs
T-cell zone	T cells	CD3, CD8, CD4	(34)
	T_FH_ cells	PD1, CXCR5	(22, 35)
	T_H_1 cells	T-bet	(36, 37)
	T_reg_ cells	FOXP3	(37, 38)
	DCs	DC-LAMP (CD208), CD83, CD86	(33)
	FRCs	CCL19, CCL21	(39, 40)
B-cell zone	B cells	CD20, CD19	(13)
	GCs	Ki67, AID, BCL-6	(13, 41, 42)
	Plasma cells	CD138, CD269	(15)
	FDCs	CD21, CD35, CD23	(20, 43)
Others	HEVs	PNAd, MECA79, CD62L	(20, 44–46)
	Macrophages	CD68, CD163	(47, 48)
	Neutrophils	CD66b, myeloperoxidase	(37, 49)

T_FH_, follicular helper T cells; T_H_1, T helper 1 cells; T_reg_, regulatory T cells; FRCs, fibroblastic reticular cells; FDCs, follicular dendritic cells; HEVs, high endothelial venules; GCs, germinal centers.

Fibroblastic reticular cells (FRCs) are specialized stromal cells localized in the T-cell compartment of TLSs that provide structural support for the normal physiological activities of immune cells, anchor TLSs in areas of chronic inflammatory tissue, and interact with the TME to regulate T cells (39, 40, 53). In addition, TLSs contain distinct DC populations; for example, CD208^+^ mature DCs, also known as dendritic cell-lysosomal associated membrane protein (DC-LAMP)^+^ DCs (12), appear in the T-cell zone and promote the T-cell response (54). CD21^+^ follicular dendritic cells (FDCs) are found in the B-cell zone, where they establish a reticular structure and integrate into the lymphoid follicle, participating in the selection of memory B cells in the GC response and capturing and retaining antigens (20, 43). High endothelial venules (HEVs), expressing a specific type of peripheral nodal addressin (PNAd)^+^ (also called MECA-79) vessel surrounding TLSs, allow immune cells, including DCs and T, B, and T_reg_ cells, to extravasate from the circulation to the periphery for entry into the tumor and are involved in lymphocyte recruitment *via* the secretion of chemokines (20, 44). Additionally, CD68^+^ macrophages dispersed within TLSs, also called tingible body macrophages, function as the exclusive professional scavengers for apoptotic cells (55–57). Finally, neutrophils are occasionally found in TLSs and may be associated with tumor metastasis and immunosuppression (37, 49).

## 3 The driving force behind the formation of TLSs

The exact mechanisms driving TLS generation in various etiological conditions, particularly cancer, are still unclear. To better understand the process of TLS formation, inspiration can be drawn from the development of SLOs. The initial understanding of prenatally formed SLOs came from the discovery that lymphotoxin-α-deficient mice do not have lymph nodes or Peyer’s patches (58, 59). The formation of programmed SLOs involves the activation of lymphotropic signaling resulting from interactions between hematopoietic and non-lymphoid stromal cells in mouse and human embryos. In addition, various molecular components, such as cytokines, chemokines, adhesion molecules, and survival factors, play key roles in the formation and localization of lymphoid structures (59, 60). Canonical SLOs, including the spleen, lymph nodes, adenoids, appendix, and mucosa-associated lymphoid tissues (MALTs), are strategically distributed throughout the body in distinct, predetermined anatomical locations, allowing lymphocytes to efficiently encounter and process antigen-presenting cells (APCs) from a variety of tissues and initiate adaptive immune responses to fight diseases (59, 60). In considering the prenatal generation of lymph nodes and Peyer’s patches and the postnatal generation of MALTs, such as bronchial-associated lymphoid tissue (BALT), we hypothesize that the development of lymphoid tissue is continuous, starting from SLOs, which are induced by genetic factors early in life, to the later formation of highly inducible and transient lymphoid structures in response to chronic inflammation (33). Thus, a possible explanation for the formation of TLSs is that the sustained chronic inflammatory environment may increase the chance of seeding lymphoid structures outside the lymph nodes, causing lymphocyte aggregation in the lesion sites (61).

The first stage of SLO development involves the production of interleukin-7 receptor alpha chain positive (IL-7Rα^+^) fetal liver precursors, which probably hinges on the expression of IKAROS, a hemopoietic-specific zinc finger transcription factor that plays a key role in regulating lymphocyte differentiation during fetal development (62). Subsequently, these IL-7Rα^+^ lymphoid progenitors differentiate into CD45^+^CD4^+^CD3^-^ lymphoid tissue-inducer (LTi) cells in a process that requires transcription factors, such as helix-loop-helix protein inhibitor of DNA binding 2 (ID2) (63), retinoic acid receptor-related orphan receptor-γt (RORγt) (64, 65), and tumor necrosis factor (TNF)-related activation-induced cytokine (TRANCE) (66). The second stage of SLO development involves the early colonization of hematopoietic LTi cell aggregates, which express the lymphotoxin-β receptor (LTβR) ligand lymphotoxin-α1β2 (LTα1β2). LTα1β2 interacts with LTβR expressed on lymphoid tissue organizer (LTo) cells—a type of stromal organizer cell that later differentiates into FDCs and FRCs. This interaction consequently leads to the next step of SLO development, namely, the upregulation of adhesion molecules. For instance, the expression of vascular cell adhesion molecule 1 (VCAM1), intercellular adhesion molecule 1 (ICAM1), mucosal vascular addressin cell adhesion molecule 1 (MAdCAM1), and PNAd, as well as homeostatic chemokines such as CCL19, CCL21, and CXCL13, is upregulated (60, 61). These adhesion molecules and chemokines are necessary not only for the attraction and retention of additional stromal and hematopoietic cells aggregating into the developing lymphatic niche (67–69) but also for the vascularization of HEVs (68, 70–72), through which immune cells can extravasate into the bloodstream. Finally, partitioning of nascent lymphoid follicles into distinct B- and T-cell areas is mediated by these chemokines and their receptors, including CXCL13 (expressed in FDCs) and CCL19 and CCL21 (expressed in the HEVs and FRCs). CXCL13 attracts CXCR5-expressing B cells and establishes lymphoid follicles, whereas CCL19 and CCL21 attract CCR7-expressing T cells and DCs, thus facilitating the formation of B- and T-cell compartments, respectively (67, 71, 73, 74).

**Figure 1:**
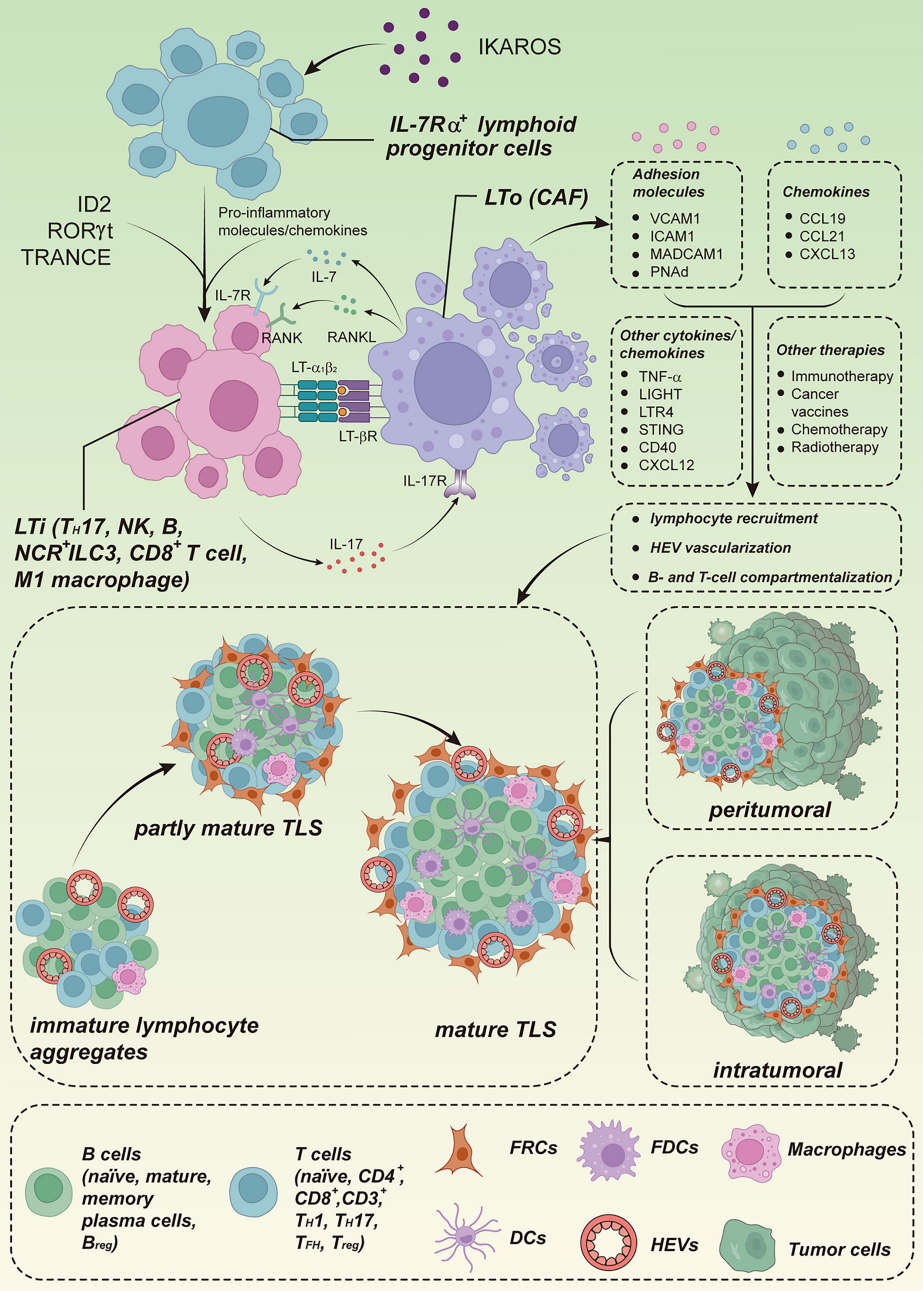
Model of TLS formation. The formation of TLSs shares similarities with the formation of SLOs. The transcription factor IKAROS regulates the production of IL-7Rα^+^ lymphoid progenitor cells, which regulate the differentiation of LTi cells using ID2, RORγt, and TRANCE. Local accumulation of pro-inflammatory molecules and chemokines promotes the recruitment of LTi cells to sites of inflammation, and aggregated LTi and LTo cells act through a combination of signaling pathways such as LTα1β2/LTβR, IL-7/IL-7R, RANK/RANKL, and IL-17/IL-17R, leading to the secretion of adhesion molecules and chemokines, which in turn promote lymphocyte recruitment, vascularization of HEVs, and B- and T-cell compartmentalisation. Some locally aggregated immune cell populations, such as T_H_17 cells, NCR^+^ ILC3 cells, M1-polarized macrophages, effector CD8^+^ T cells, NK cells, and B lymphocytes, have the potential to functionally replace LTi cells. Some local stromal and immune subsets also have the potential to replace LTo cells, such as CAFs. In addition, other cytokines/chemokines (TNF-α, LIGHT, TLR4, STING, CD40, CXCL12) and therapeutic regimens (immunotherapy, cancer vaccines, chemotherapy, radiotherapy) can also induce TLS. TLSs can be located intratumorally and/or peritumorally, and the maturity of TLSs progress through immature lymphocyte aggregates to partially mature TLSs with FDCs, but no GC, and finally to mature structures with a GC. IL-7Rα, interleukin-7 receptor alpha chain; RANKL, receptor activator of nuclear factor kappa-B ligand. Cited and adapted from a published figure from the article “T. N Schumacher, D. S. Thommen, Tertiary lymphoid structures in cancer. Science (2022).”, Copyright ^©^ 2022, The American Association for the Advancement of Science.

While SLOs typically arise at target sites under the precise control of developmental programs, TLS formation in tumors occurs randomly after birth, usually in non-lymphoid locations, and may vary by tumor type. A model for TLS formation is shown in **Figure 1**. The tumor-associated process of TLS neogenesis is characterized by the generation of anatomically similar, organized lymphocyte aggregates in the context of chronic immune activity but lacking a surrounding capsule membrane (70, 75, 76). The absence of a capsule may reflect the manner in which TLSs arise, and the order in which these stromal cells and other organizer cells populate TLSs may differ from the precise spatiotemporal regulation of SLOs, even though most of these elements are also present in TLSs (77). The general absence of the capsule may also have implications for trafficking, allowing cellular components of TLSs to translocate directly into the surrounding tissue to fight disease, whereas in lymph nodes, DCs and T cells must migrate through the surrounding medullary sinus into the parenchyma (78).

Furthermore, TLSs may be generated *via* induction signals that are distinct from those involved in SLO formation. Most of our knowledge of TLS formation comes from transgenic mouse models of autoimmune diseases and chronic infections that ectopically express various chemokines and cytokines; therefore, the findings from these disease models can only serve as a hypothesis for the formation of TLSs in the context of cancer. For example, the role of LTi cells in the evolution of TLSs remains an important unanswered question. Using CCL21 transgenic mice hybridized with ID2^-/-^ mice, which are devoid of LTi cells, researchers found that TLSs can form in the thyroid; however, no canonical SLOs were found, suggesting that TLSs can be induced independently of ID2-dependent LTi cells (61, 79). Several locally clustered immune cell populations have been predicted to replace LTi cells, including T helper cells secreting IL-17 (T_H_17) (75, 80), natural cytotoxicity receptor (NCR)^+^ innate lymphoid cells (NCR^+^ILC3) (81), M1-polarized macrophages (82), effector CD8^+^ T cells and NK cells (83), and B lymphocytes (84). Such cell populations have been demonstrated to interact with stromal cells to initiate TLS formation when LTi cells are absent in various pathological contexts, including cancers. One reason why LTi cells may not be required for the development of TLSs is that these tissues usually form later in life in response to chronic inflammatory or infectious conditions. Because adults have a broad range of circulating lymphocytes expressing LTαβ, these cells may function in place of LTi cells when activated (85, 86). It is also noteworthy that the induction of TLS formation may not always be dependent on lymphotoxin production by LTi cells, as the inducible BALT (iBALT) model (a type of TLS formed in lung tissue generated by repeated intranasal sensitization to lipopolysaccharide in neonatal mice) was reported to be dependent on IL-17 production by CD4^+^ T cells and triggered by IL-17R^+^ stromal cells expressing CXCL13 and CCL19 (87). Not coincidentally, normal iBALT regions were also observed in LTi^-/-^ mice, where T and B cells aggregated but were not segregated into distinct regions, FDCs were not formed, and HEVs expressing PNAd were not detected; thus, further discernment of whether these iBALT regions are bona fide TLSs may be required (88). Interestingly, some local stromal and immune subsets also have the potential to replace LTo cells in promoting TLS formation; for instance, the development of tumor-associated tertiary lymphoid structures (TA-TLSs) in mouse melanoma is orchestrated by cancer-associated fibroblasts (CAFs) with LTo cell characteristics that are induced by TNF receptor signaling (39). According to the Guedj’s group, secretion of chemokines by vascular smooth muscle cells and mesenteric adipocytes stimulated by inflammatory signals induced the recruitment of immune cells and functional TLS formation in atherosclerotic aorta and Crohn’s disease-affected mesentery, respectively (89, 90). These stromal cells were predicted to function similar to LTo cells in promoting TLS development and/or sustainment.

## 4 Function of TLSs in tumor immune responses

**Figure 2:**
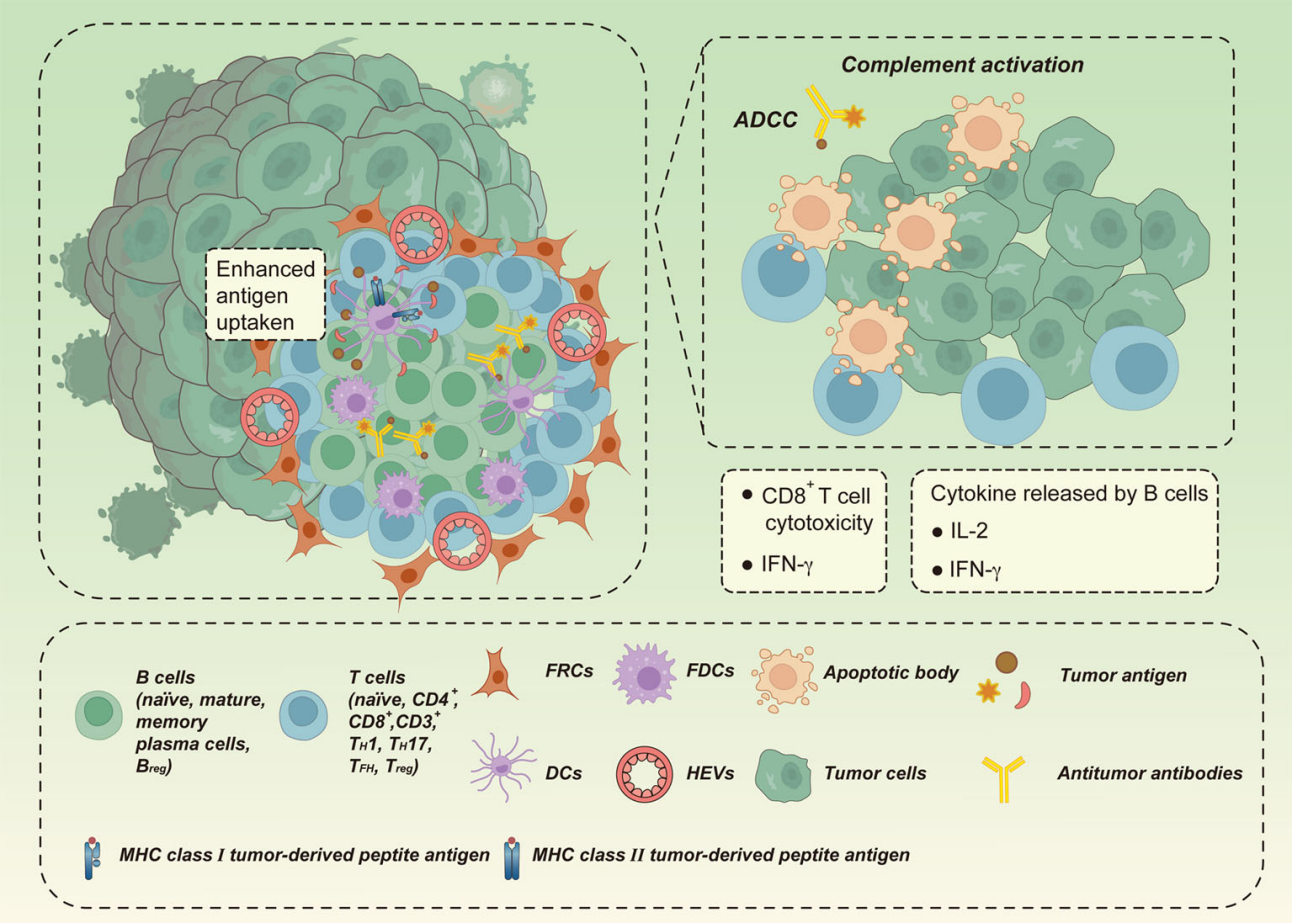
The antitumor effect of TLSs. TLSs may enhance local immune responses at the site of tumor-associated TLS (TA-TLS) formation by B cells directing T cells to recognize tumor-associated antigens (TAAs) to clear infection or kill tumors. B cells may trigger T cell initiation by presenting captured TAAs directly to T cells *via* B cell receptors. B cells may also produce antibodies against TAAs locally by acting in coordination with CD8^+^ cytotoxic effector T cells. Tumors are eliminated *in situ* through direct killing of tumor cells, antibody-dependent cytotoxicity (ADCC) mediated by macrophages and/or natural killer cells, and local complement activation. In addition, B cells can drive cytotoxic immune responses by releasing cytokines (e.g., IFN-γ and IL-12), thus promoting antitumor immunity.

Considering the anatomical similarities, we can infer that the function of TLSs may also be similar to that of SLOs. SLOs, especially lymph nodes, are essential for accelerating the initiation and execution of specific immune responses, as they provide specialized niches for the encounter of antigen-specific lymphocytes from the circulation and APCs from locally infected or inflamed tissues, thus increasing the contact between immune cells and ensuring rapid immune response initiation (70, 91). Similarly, the underlying mechanisms of TLSs have been investigated and found to mainly enhance the local immune response at the site of TA-TLS formation through B cells instructing T cells to recognize TAAs (56, 92) (**Figure 2** **)**. This heightened local immunity could lead to clearance of infection or rejection of a tumor. B cells expressing MHC II can directly present captured TAAs to CD4^+^/CD8^+^ T cells *via* B-cell receptors, thereby triggering T-cell priming (93, 94). B cells can also perform antitumor functions through the local generation of antibodies targeted to TAAs; these secreted antibodies subsequently activate downstream adaptive immune responses and induce cancer-immune cycles locally through several mechanisms, including antibody-dependent cytotoxicity of NK cells, phagocytosis by macrophages, antigen presentation by DCs, and local complement activation (93, 94). Activation-induced cytidine deaminase (AID) was detected in the TLS-GCs of non-small cell lung cancer (NSCLC) and found to support local autoantibody production and the occurrence of clonal expansion, somatic hypermutation, and homotypic switch in B cells (41, 95); Similarly, BCL-6, a transcription factor involved in late stages of B-cell maturation, was found in TLS-GCs in oral squamous cell carcinoma. These studies provide evidence that B-cell clonal proliferation, isotype switching, and effector B-cell differentiation actively occur in TLSs (41, 95). Additionally, B cells may facilitate antitumor immunity by releasing cytokines, such as IFN-γ and IL-12, that act as drivers of cytotoxic immune responses (96). Collectively, these data provide compelling evidence that TLSs are capable of reproducing SLO function locally. The coordinated action of T and B cells generated in TLSs allows *in situ* destruction of tumors, improving prognosis or response to immunotherapy in patients with certain tumors, including breast cancer, NSCLC, ovarian cancer, colorectal cancer (CRC), liver cancer, melanoma, and soft tissue sarcomas (19, 41, 97).

It remains unclear whether TLSs are primarily used to educate T cells or to initiate naïve T cells for better tumor control. Several investigators have suggested that TLSs can educate tumor-infiltrating lymphocytes to fight tumors. Patients with both TLSs and high CD8^+^ T-cell infiltration exhibited significantly improved survival relative to patients with high CD8^+^ T-cell infiltration without TLSs, suggesting the prognostic value of TLSs to actively license intra-tumoral cytotoxic T cells (30). Similarly, the presence of plasma cells expressing antigen-specific response markers within TLSs in ovarian cancer has also been linked to an increased response by tumor-infiltrating CD8^+^ T cells (19). Importantly, in NSCLC, the presence of mature TA-TLSs containing DCs was associated with T-cell activation, T_H_1 phenotype, and cytotoxic orientation, further supporting the idea that TLSs may play a role in shaping the immune profile of the TME by educating T cells (47). However, the latter hypothesis that TLSs initiate naïve T cells is supported by the finding in a mouse model of multiple sclerosis that naïve T cells are recruited into TLSs in the inflamed CNS and activated by local APCs (possibly DCs) to initiate epitope spreading (98). Likewise, the expression of LIGHT (also known as tumor necrosis factor ligand superfamily member 14, TNFSF14) in fibrosarcoma cell lines upregulates CCL21 and MAdCAM1 on the tumor vasculature and favors the recruitment of massive numbers of naïve CD8^+^ T cells, followed by full activation *in situ* to promote rejection of established, highly progressive tumors (99). HEVs found in human and mouse tumors, whether associated with TLSs or not, express adhesion molecules and chemokines (e.g. PNAd, MAdCAM1, CCL21, and ICAM1) that may contribute to their interaction with naïve and central memory lymphocytes, facilitating their drainage from the blood and recruitment into tumor tissues (83, 100–103). In a preclinical mouse model of colon carcinogenesis, administration of GFP-labelled splenocytes intravenously resulted in the homing of lymphocytes to TLSs in the colonic mucosa, implicating an active role of TLSs in recruiting lymphocytes to the tumor region (20).

Although the significance of the process of replicating nascent lymphoid tissue at the tumor site is still poorly understood, Schumacher et al. first proposed four models, namely, speed, efficiency, control, and survival, to help explain the advantages of chronic inflammatory stimuli leading to the establishment of lymphatic niches at sites of infection or cancer (31). Local creation of a lymphatic niche may enhance antitumor immunity in different ways, allowing for: 1) direct initiation of T and B cells at the lesion site, thus saving the time needed for DCs and lymphocytes to enter and exit SLOs; 2) increased chances of lymphocytes encountering similar antigens; 3) direct infiltration of cytokines from the TME into the envelope-free TLS; and/or 4) the ability to screen for dominant lymphocytes with preferential tumor responsiveness that are resident at the tumor site.

## 5 TLSs as biomarkers for predicting prognosis and therapeutic response in cancers

Given the regulatory role of TLSs in tumors, an increasing number of researchers have evaluated their potential impact on clinical outcomes in various cancers. In fact, the presence of TLSs is associated with patient survival and response to therapy. Hence, TLSs may become valuable biomarkers for predicting prognosis or monitoring therapeutic responses.

### 5.1 Prognostic associations

As mentioned previously, the main function of TLSs in tumors is to enhance the adaptive immune response at the site of formation, which contributes to the clearance of infection or rejection of tumors and improves the prognosis of most cancer patients (15), including those with intrahepatic cholangiocarcinoma (iCCAs) (104), lung cancer (33), endometrial cancer (105), CRC (106), and pancreatic cancer (107). However, a wide variety of biomarkers is used to identify TLSs, implying that TLSs are heterogeneous across cancer types and patients. Accordingly, it is hypothesized that the location, density, components, and maturation stages of TLSs in tumors, as well as the stage and location of the TLS-forming tumor, may correlate with positive or negative prognosis of patients (**Table 2**).

**Table 2:** TLSs as biomarkers in cancers.

Prognostic associations								
TLS features		Cancer types	Percentage of positive TLS	Method of TLS detection	Predictive value		Refs	
Locations	Intra-tumoral	iCCAs	578/962	IHC	Positive		(25, 99, 103, 104)	
		HCC	129/173	H&E				
		Breast cancer	148/167	IHC				
	Peritumoral	iCCAs	815/962	IHC	Negative		(25, 99, 103, 104)	
		HCC	N.A.	IHC				
		Breast cancer	41/167	IHC				
Components	DC-LAMP^+^ DCs	NSCLC	52/74	IHC	Positive		(92)	
	MECA-79^+^ HEVs	Breast cancer	74/146	IHC/IF	Positive		(108)	
	T_FH_ cells	Breast cancer	35/70	IHC	Positive		(35)	
	Follicular B cells	NSCLC	90/196	IHC	Positive		(88)	
	T_reg_ cells	Lung adenocarcinoma	N.A.	IHC/IF	Negative		(109, 110)	
	B_reg_ cells	HCC	47/74	IHC	Negative		(111) (112) (113) (114)	
		TSCC	N.A./46	IHC				
		Gastric cancer	44/59	IHC				
		Breast cancer	79/125	IHC				
Maturation	Mature	HCC	N.A.	N.A.	Positive		(13)	
	Immature	HCC	30/127	IHC	Negative		(101, 115)	
		CRC	106/109	IF				
Tumor status	Primary	Melanoma	N.A.	IHC	N.A.		(116, 117)	
		Breast cancer	60/160	H&E	N.A.			
	Metastatic	Melanoma	9/27	IHC/ICM	N.A.		(116–118)	
		RCC and CRC lung metastases	140/192	IHC	CRC positive, RCC negative			
		Breast cancer lung metastases	48/160	H&E	Positive			

Therapeutic responses								
Therapies		Cancer types	Percentage of positive TLS	Method of TLS detection		Predictive value		Refs
Immunotherapy (anti-CTLA-4/PD-1/PD-L1)		Melanoma	N.A.	RNA-seq/ IHC/ IF		Positive		(21, 119–125)
		Soft-tissue sarcoma	11/73	IHC/ IF				
		Urothelial carcinoma	10/19	IHC/ IF				
		NSCLC	7/20	H&E				
		RCC	18/24	Spatial transcriptomics/ IF				
		Pancreatic cancer	105/328	IHC/ IF				
Chemotherapy		Squamous cell carcinoma	N.A.	IHC		Negative		(126, 127),
		Breast cancer	100/368	IF		Positive		
Radiation therapy		Solid tumours	N.A.	12-CK		N.A.		(128, 129)
Targeted therapy		Metastatic melanoma	11/16	IHC		N.A.		(130)

Several studies have reported that TLSs distributed in different parts of the tumor may signify distinct outcomes for patients. In iCCAs, the presence of intra-tumoral TLSs (which are negatively linked to tumor diameter, neoplasm necrosis, and microvascular infiltration) was significantly associated with more favorable overall survival than the presence of peritumoral TLSs (which are positively related to lymph node metastasis and high serum gamma-globulin transferase and carbohydrate antigen 19-9 levels) (104). Thus, opposing prognostic values and different clinicopathological correlations are associated with TLSs in distinct areas, suggesting a fundamental difference in the local immune milieu of the tumor. Patients characterized by a large number of intratumoral TLSs and very few peritumoral TLSs experience a strong antitumor immune response and have good prognosis; while the opposite indicates immunosuppression and poor prognosis (104). This dual prognostic value of TLSs based on spatial differences has also been identified in HCC (25, 108) and breast cancer (34), and the underlying mechanism may involve different cellular compositions of TLSs within and around tumors, which may determine their pro-tumor or antitumor activities. Notably, however, a clear association between the spatial location of TLSs and prognosis has not been identified for most cancers, further indicating high TLS heterogeneity in different cancers.

TLS densities and cellular composition (e.g. DC-LAMP^+^ mature DCs, MECA-79^+^ HEVs, T_FH_ cells, and follicular B cells) have been shown to correspond to improved survival rates in patients with different tumor types (1, 7, 107, 119, 120, 130). TLSs were first reported in solid tumors in a retrospective study of 74 patients with early-stage NSCLC, called Ti-BALT, and consisted of an organized distribution of mature DC/T-cell populations and B-cell follicles; the high density of mature DC-LAMP^+^ (CD208^+^) DCs, a hallmark of Ti-BALT, was associated with favorable clinical outcomes (47). Interestingly, in a retrospective cohort of 146 patients with invasive breast cancer, high-density tumor MECA-79^+^ HEVs localized exclusively in lymphocyte-rich areas were found to independently predict a lower risk of recurrence, and were significantly associated with longer metastasis-free, disease-free, and overall survival (131). This finding may be explained by the key role that HEVs play in locally enhancing antitumor immunity by serving as gateways for recruiting naïve T and B cells into the tumor and the subsequent formation of TLSs (121, 131). Regarding T_FH_ cells, Gu-Trantien and colleagues showed that in breast cancer, CXCL13-producing T_FH_ cells located in the GC of TLSs strongly predicted favorable survival or preoperative response to chemotherapy (51). In addition, a high density of follicular B cells in TLSs was found to be associated with long-term survival in patients with early-stage or advanced NSCLC treated with chemotherapy (41). Altogether, these findings strongly suggest that certain components of TLSs may serve as biomarkers of positive prognoses for a subset of tumors.

It is noteworthy that the presence of T_reg_ and regulatory B (B_reg_) cells has been observed in TLSs in breast tumors, prostate cancer, lung squamous cell carcinoma, and pulmonary metastases from CRC and bladder cancer (37, 51, 112, 122–124), and their high densities have been related to adverse clinical outcomes, suggesting that T_reg_ and B_reg_ cells have an immunosuppressive effect on these ectopic lymphoid tissues. In a mouse model of lung adenocarcinoma, depletion of T_reg_ cells resulted in tumor destruction, suggesting that T_reg_ cells in TA-TLSs can suppress endogenous tumor-specific immune responses (132, 133). In a mouse model of fibrosarcoma, the presence of T_reg_ cells was thought to impede TLS development by blocking HEV induction and immune infiltration (103, 125). Furthermore, in patients with bladder cancer, high numbers of IL-10^+^ B_reg_ cells were associated with shorter overall survival (112), and this unfavorable prognosis was also reported in HCC (134), tongue squamous carcinoma (109), gastric cancer (112), and breast cancer (110). These findings indicate that B_reg_ cells residing in TLSs may also suppress tumor-specific immunity, perhaps *via* IL-10 secretion in the TME (13, 94), which may impair the effector function of T cells, ultimately resulting in an ineffective antitumor immune response. Importantly, there is no direct evidence that “suppressive” TLSs promote tumorigenesis, but localized tumor-specific alterations in the properties of TLSs may provide an effective means to address this issue. A detailed analysis of the expression of immune checkpoint molecules in tumor-infiltrating immunoregulatory cells and the study of individual TLSs during tumor progression are important future directions.

The consecutive maturation stages of TLSs within a tumor (**Figure 1**), from the immature stage (dense lymphoid aggregations without FDC networks) followed by a partly mature TLS (with FDCs but no GC response) to a fully mature TLS (separation of T and B lymphocytes into two distinct regions with an active GC response), have been distinguished. The presence of immature TLSs in the preneoplastic lesions of HCC (135) and CRC (106) has been associated with an elevated incidence of cancer recurrence. Thus, this very early stage of TLS formation appears to be detrimental to effective anti-neoplastic immunity, likely owing to the increased expression of immunosuppressive molecules. Instead, a highly organized TLS may be required to achieve the complex architecture required for optimal interactions between various immune cell types to facilitate an effective immune response. The observation that the intra-tumoral and peritumoral GC-containing, mature TLS correlates with longer recurrence-free and overall survival in patients with HCC illustrates this point clearly (13).

Finally, the antitumor effect of TLSs may be affected by the stage and location of the tumors themselves. Cipponi et al. found that *de novo* and antibody responses to ectopic lymphoid structures were observed more often in metastatic lesions than in human primary melanoma, although they did not assess the prognosis of patients in this cohort (111). Interestingly, with comparable densities of primary and corresponding metastases, TLSs were mostly immature and associated with poor survival in pulmonary metastases from renal cell carcinoma (RCC), whereas they were more mature and associated with positive outcomes in pulmonary metastases from CRC (114). However, another study reported the opposite result that high-density TLSs corresponded with poor outcomes in primary breast cancer but improved clinical outcomes in metastatic lesions (113). In conclusion, these data emphasize the importance of the tumor origin for molding a specific tumor immune microenvironment and determining TLS neogenesis. We speculate that the TME is altered when the primary tumor metastasizes at an advanced stage and established immunosuppressive mechanisms affect the immune function of TLSs.

### 5.2 Associations with therapeutic responses

A growing body of data suggests that cancer therapies that stimulate antitumor immune responses are more effective in generating long-term responses (7, 136, 137). Such therapies include immunotherapy (138, 139), conventional chemotherapy (140), radiation therapy (128, 141), and targeted therapies (129). TLSs, as important components of the tumor immune environment (137), are the primary sites for generating antitumor immune responses (14, 16); thus, it is necessary to assess their impact on therapeutic responses and understand how they are modulated by treatment.

Specifically, the presence of TLSs and B-cell infiltration has been shown to help predict patient responses to programmed death-1 (PD-1) monotherapy or combined blockade with PD-1 plus cytotoxic T-lymphocyte-associated protein 4 (CTLA-4) in pre-therapeutic biopsy specimens of melanoma, soft-tissue sarcoma, urothelial carcinoma, NSCLC, and RCC (21, 115–118, 142, 143). In particular, a recent study of RCC-associated TLSs by Meylan et al. using formalin-fixed paraffin-embedding spatial transcriptomics showed that patients with TLS-positive tumors exhibited higher remission rates and longer progression-free survival with ICB treatment. Mechanistically, this may be explained by intra-tumoral TLSs maintaining B-cell maturation and antibody production, which may lead to a complete B-cell response inside the tumor, resulting in a direct antitumor effect (117). Importantly, these B cells may also contribute to the therapeutic efficacy of ICB by altering T cell activation and function, as well as by acting with other key immune components of TLSs through other mechanisms; thus, TLSs may serve as predictors of ICB responsiveness (142). In addition, a gene signature related to the presence of TLSs can predict patient responses to anti-CTLA4 and anti-PD-1 antibodies, either alone or in combination (115). A pan-tumor study of three independent cohorts of over 500 patients with advanced cancer treated with anti-PD-1 or anti-PD-L1 antibodies revealed that the presence of mature TLSs, identified using immunohistochemical co-staining for CD3, CD20, and CD23, predicted immunotherapy efficacy and improved progression-free and overall survival regardless of PD-L1 expression (144). Notably, the value of TLSs as predictors of the response to ICB does not appear to be tumor type-specific, as similarly favorable outcomes have been observed in tumors considered insensitive to ICB, such as pancreatic cancer and sarcoma (144).

Clinical application of these findings can improve patient selection for immunotherapy because standard pathology laboratories are able to rapidly identify TLSs in patients. Prospective studies using TLSs for the selection of immunotherapy candidates were published in 2022 (145). Researchers selected patients with advanced soft tissue sarcoma (a type of tumor with limited sensitivity to current ICB) for anti-PD-1 therapy based on the presence of TLSs in tumor biopsies and found a substantial improvement in the overall response rate and median progression-free survival in the TLS-positive cohort compared to the previously unselected cohort. These results highlight the potential for TLS testing to select sarcoma patients who are more likely to respond to ICB (145). Hence, multiple clinical trials examining ICB (including NCT04705818 and NCT03475953) are implementing TLSs as an inclusion criterion for applicant selection, with results expected in the near future.

Intriguingly, the formation of TLSs was found to be facilitated by ICB treatment based on the analysis of tumor biopsies performed prior to treatment. In a single-arm feasibility trial (ClinicalTrials.gov: NCT03387761), 24 patients with stage III uroepithelial carcinoma received neoadjuvant anti-PD-1 therapy combined with CTLA-4 blockade followed by resection, and post-treatment induction of TLS formation was observed in tumor specimens from responding patients (126). In another cohort of patients receiving neoadjuvant anti-PD-1 therapy for NSCLC, TLS enrichment was also observed in resected specimens from post-treatment responders (21). Likewise, in a melanoma mouse model, immunotherapy was found to induce increased numbers and sizes of TA-TLSs, which typically consisted of discrete T- and B-cell areas; the presence, quantity, and magnitude of TA-TLSs corresponded with improved tumor control and overall response to checkpoint immunotherapy (127). Although many findings support the model that ICB treatment influences TLS function (142, 146, 147), direct evidence is still lacking as this field of research is currently in its infancy. Notably, while TLS-rich tumors experienced higher infiltration by CD8^+^ T cells, these T cells may be dysfunctional or exhausted after prolonged exposure to relevant antigens (116). However, ICB may activate T cell function by blocking immune checkpoint proteins, such as PD-1, PD-L1, and CTLA-4, which explains why ICB may enable TLS-rich tumors to develop antitumor immunity (116). Future investigations are required to improve our understanding of the ICB-mediated regulation of TLS formation; for instance, ex vivo assessment of ICB-treated human tumor cultures (148) as well as spatial analysis of neoplasms at very early time points after treatment initiation will aid in understanding this phenomenon.

In addition to immunotherapy, TLSs may have an impact on therapeutic responses to conventional chemotherapy (140). For example, in a mouse model of squamous cell carcinoma, depletion of B cells in ectopic lymphoid structures using the anti-CD20 antibody rituximab improved the response to chemotherapeutic agents (149). Furthermore, a subset of B cells expressing inducible T-cell costimulator ligand (ICOS-L) appeared after neoadjuvant chemotherapy in breast cancer patients and aggregated in the intra-tumoral TLS; this process triggered a T-cell response as part of the antitumor response and was associated with favorable clinical outcomes in these patients (150). Together, these data suggest an interaction between TLSs and chemotherapeutic responses, although different cell populations may have opposing effects.

## 6 Therapeutic induction of TLSs

Therapeutic approaches involving the induction of TLSs are currently used in cancer treatments and are increasingly seen as a promising strategy for cancer therapy (**Table 3**). In mouse models, local TLSs can be induced by the expression of TLS-related cytokines or chemokines, such as lymphotoxin (151), TNFα (152, 153), LIGHT (154), stimulator of interferon genes (STING) (155, 156), Toll-like receptor 4 (TLR4) (88, 157), CD40 (13), CXCL13 (74), CCL19 (158), CCL21 (158), and CXCL12 (158). Additionally, anti-PD-1 treatment of NSCLC in an immunocompetent mouse model revealed that circulating T_FH_ cells are associated with enhanced B-cell activation capacity, increased CCL21 levels, increased number of TLSs in tumors, and the production of antitumor antibodies *in situ* that interfere with tumor growth (147). Additionally, in mouse models of breast and pancreatic neuroendocrine tumors, TLSs were shown to be therapeutically induced and associated with tumor control; anti-angiogenic therapy plus PD-L1 blockade could modulate the angiogenic vasculature, induce TLSs, and enhanced cytotoxic T-cell activity, with ensuing survival benefits (159, 160).

**Table 3:** Strategies for inducing TLS formation in cancer-bearing hosts.

Therapies	Contents	Cancer type	Maturity of induced TLS	TLS detection	Prognostic value	Refs
TLS-related cytokines/ Chemokines	Lymphotoxin	N.A.	Lymphoid aggregates	H&E	N.A.	(151)
	TNFα	N.A.	Lymphoid aggregates with DCs	IF (CD3, B220, PNAd)	N.A.	(152, 153)
	LIGHT	N.A.	Lymphoid aggregates	IHC (CD3, B220, MAdCAM-1)	N.A.	(154)
	STING	Melanoma	Lymphoid aggregates with DCs and HEVs	IHC (CD3, CD11c, PNAd)	N.A.	(155, 156)
	TLR4	N.A.	GC-containing	IF (CD3, CD21, B220, PNAd)	N.A.	(88, 157)
	CD40	Glioblastoma	GC-containing	IF (CD45, B220, CD21, CD35)	N.A.	(13)
	CXCL13	N.A.	GC-containing	IF (CD3, CD11c, PNAd)	N.A.	(74)
	CCL19	N.A.	Lymphoid aggregates with DCs and HEVs	IHC (CD3, CD4, CD11C, PNAd)	N.A.	(158)
	CCL21	N.A.	Lymphoid aggregates with DCs and HEVs	IHC (CD3, CD4, CD11C, PNAd)	N.A.	(158)
	CXCL12	N.A.	Lymphoid aggregates with DCs and HEVs	IHC (CD3, CD4, CD11C, PNAd)	N.A.	(158)
Immunotherapy	Anti-PD-1/PD-L1	NSCLC	GC-containing	IHC (PD-1, Ki67)	N.A.	(147, 159, 160)
	Anti-CTLA4	Melanoma colorectal cancer	GC-containing	IF (CD20, CD21, PNAd, DC-LAMP)	Positive	(126)
Cancer vaccines	HPV vaccines	CIN2/3	GC-containing	IHC (CD8, CD20, PNAd, Foxp3, Ki67)	Positive	(161)
	GVAX	PDAC	GC-containing	IHC (CD20)	Positive	(162)
Chemotherapy	Neoadjuvant chemotherapy	PDAC	GC-containing	IHC (CD20, PNAd, Ki67, Foxp3)	Positive	(48, 150, 163, 164)
Radiotherapy	Hypo-fractionated radiation	Breast cancer	GC-containing	IHC (CD20, Foxp3), FCM (granulocytes)	N.A.	(165)

LIGHT, also called TNFSF14, tumor necrosis factor ligand superfamily member 14; STING, stimulator of interferon genes; TLR4, Toll-like receptor 4; IHC, immunohistochemistry; FCM, flow cytometry; PDAC, pancreatic ductal adenocarcinoma; CIN2/3, cervical intraepithelial neoplasias; IF, immunofluorescence; H&E, hematoxylin-eosin staining; N.A., not available.

Emerging evidence suggests that some standard chemotherapy regimens (such as oxaliplatin) promote anticancer immune responses by inducing immunogenic cell death, leading to immune reconstitution accompanied by the formation of intra-tumoral TLSs and B-cell infiltration (166). Based on a retrospective study, tumors from pancreatic ductal adenocarcinoma (PDAC) patients who were treated with neoadjuvant chemotherapy contained more CD8^+^ T cells, PNAd^+^ HEVs, CD163^+^ macrophages, and Ki67^+^ cells than those from patients who received upfront treatment (48). In a small cohort of children with hepatoblastoma germline APC mutations, pathologic review identified substantial intra-tumoral TLSs with both lymphocytes and APCs in all tumors receiving cisplatin-based neoadjuvant chemotherapy, but not in pre-chemotherapy samples, suggesting that these TLSs may have been induced by chemotherapy (163). According to Lu et al., neoadjuvant chemotherapy in breast cancer patients induced ICOS-L^+^ B cells expressing complement receptor CR2, which may lead to TLS induction and improved disease-free and overall survival (150). A significant association between neoadjuvant chemotherapy and TLSs was also observed among 138 patients with epithelioid mesothelioma, even though the presence of TLSs did not appear to be prognostic (164). These findings suggest that some chemotherapy treatments can induce TLS formation, which may play a key role in re-establishing the immune population within tumors that was destroyed by chemotherapy. Future work comparing the TLS induction capacity of different chemotherapy regimens will help inform treatment selection.

Radiotherapy, a widely used cancer therapy, was found to induce intra-tumoral TLSs in a Kras^LSL-G12D/WT^; p53^FL/FL^-driven lung cancer mouse model (that develops spontaneous tumors (150, 167)) owing to its ability to kill local immune cells (165). In this study, hypo-fractionated radiation initially caused acute immune cell depletion and reduced the pre-existing TLS size, which was restored two weeks later (165). Further studies are needed to determine whether radiotherapy induces intra-tumoral TLS formation in cancer patients.

TLS formation is also observed when therapeutic vaccination is administered to immunocompromised patients with tumors. For instance, in patients suffering from high-grade cervical intraepithelial neoplasia, Maldonado et al. observed marked immunological alterations in the microenvironment of target lesions following intramuscular therapeutic vaccination against the HPV16 E6/E7 antigen (161). These changes included the proliferation of clonal T cells within the tumor lesions and the formation of mature TLSs in the surrounding stroma, which indicated an effector response to vaccination (161). Mechanistically, these histological changes in the stroma involve increased expression of genes related to immune activation (e.g., CXCR3) and effector functions (e.g., T-bet and IFN-β) (161). Lutz et al. compared the use of an irradiated allogeneic granulocyte-macrophage colony-stimulating factor-secreted PDAC vaccine (GVAX) as a single agent or in combination with low-dose cyclophosphamide to deplete T_reg_ cells and showed that 84.6% of patients developed intra-tumoral tertiary lymphoid aggregates (TLAs) within 14 days (162), with a morphology resembling that of TLSs. Microdissected TLA gene expression analysis revealed upregulation of signaling pathways involved in immune cell activation and trafficking, inhibition of the T_reg_ cell pathway, enhancement of the T_H_17 pathway, induction of T-cell infiltration, development of TLSs in the TME, and improved patient survival (162). Collectively, these studies suggest that intra-tumoral TLSs can be induced by cancer vaccines and correlate with increased immune effector function and beneficial clinical outcomes.

However, a key factor that has to be considered is that, although induction or enhancement of TLS function is beneficial for tumor control, the application of these intervention strategies may also promote autoreactive T and B cell responses in other parts of the body. It has been reported that despite breakthroughs in clinical oncology treatment, ICB is often accompanied by autoimmune or auto-inflammatory adverse events, termed immune-related adverse events (irAEs), with clinical manifestations primarily associated with activation of immune cells (168). irAEs have been reported in almost all organ systems and include arthritis, myositis, thyroiditis, vasculitis, and colitis (168). It is conceivable that the approach to TLS induction may also increase ICB-induced autoimmune toxicity, as TLSs have been found to support the local inflammatory process in many autoimmune diseases. It has been reported, although data are scarce, that TLS formation is associated with autoimmune myopathy after PD-1 blockade (169); biopsies from patients with myalgia and muscle weakness after anti-PD-1 treatment showed that muscle fiber damage caused by CD8^+^ cytotoxic T cells may be associated with the formation of TLS-like structures (169). Thus, induction of TLS may enhance antitumor responses but may also stimulate autoreactive T and B cell responses, and therefore, the pros and cons of this strategy need to be carefully evaluated.

## 7 Conclusions and perspectives

For researchers and clinicians, TLSs are certainly an attractive target to enhance the immune response against cancer, with the potential for improving cancer patient outcomes (**Table 4**). Among other possibilities, TLSs may be developed as a new predictive biomarker for cancer detection and response to immunotherapy.

**Table 4:** Strategies for TLS detection in tumor sections.

Contents	Strategies
Selection of tumor specimens	A tumor region containing infiltrative margins and highest immune infiltration
TLS identification	H&E staining with or without validation using multiple IHC/IF staining on serial sections Makers: CD3, CD20, PNAd, DC-LAMP Optional: CD21, Ki67
TLS Quantification	Whole section quantification of lymphoid aggregates using H&E staining Or calculating positive cells in TLSs: DC-LAMP^+^ DCs within CD3^+^ T cell enrichment zone with/without CD20^+^ B cells within B cell enrichment zone (with/without GC)

The way forward in this field involves two vital aspects. First, certain procedures and methods used in pathology laboratories need to be standardized to facilitate simple and robust detection and quantification of TLSs in tumor samples, thus improving patient selection and response assessment. Two different methods, namely, *in situ* and transcriptome analysis, have been used to detect the presence of TLSs within tumors and have yielded broadly similar results (170). Of these, *in situ* analysis may involve the staining of paraffin-embedded tumor sections with haematoxylin and eosin to achieve visual quantification of TLSs with GCs in tumor specimens (15). Moreover, immunostaining of tumor sections using the proliferation marker Ki67 allows the detection of bona fide GC B cells, and combinatorial immunostaining of CD20, CD3, and CD23 allows the detection of the FDC network within GCs, which is indicative of a mature TLS (15). With the advent of artificial intelligence (AI), analyzing microscopic images can be automated and TLSs can potentially be identified and quantified using repeated measurements of spatial components within TLSs, which may aid in the standardization of TLS-testing methods (50). AI may also allow for the automatic detection of cells of interest within tumors and the classification of immune cell subsets based on their morphology and organization, which could be used to generate a TLS score for each tumor subtype. Thus, AI will likely serve as a valuable tool for the implementation of TLS testing in translational research and clinical practice (50).

With respect to transcriptomic analysis, gene expression signatures in tumor specimens have been previously used to determine the presence of TLSs, including the 12-chemokine gene signature (171), 8-gene signature representing T_FH_ cells (51), 19-gene signature representing T_H_1 cells and B cells (172), CXCL13 signature (173), and plasma cell signature (19). However, future studies are needed to determine the optimal TLS transcriptome signature. Despite the effectiveness and specificity of immunohistochemistry in detecting TLSs in tissue sections, based on previous experience, mRNA extracted from TLS-positive cancer tissues is typically heterogeneous when compared to other characteristics. Therefore, the development of robust TLS assays using data from transcriptomic analyses of tumor samples, especially for the analysis of samples where the TME structure has not been preserved, will be beneficial for subsequent work in clinical studies.

Further investigation of TLSs in the future will require less invasive or non-invasive methods to assess biomarkers, which are particularly valuable for long-term patient monitoring to detect disease recurrence or progression, especially in cancer patients undergoing treatment or inoperable patients. The use of peripheral blood or other easily collected bodily fluids to predict and quantify the presence of TLSs is considered a promising alternative to current invasive means and will facilitate the detection of TA-TLSs and identify novel liquid biopsy markers relevant to clinical outcomes. Surprisingly, a similar concept has been explored in patients with Sjögren syndrome, in whom researchers found that ectopic GC formation in salivary gland TLSs may be mediated by pregnancy-associated plasma protein A (PAPPA), thrombochondroitin 1, and YY peptide, based on salivary proteomic analysis (174).

The second aspect in the development of TLS-associated cancer therapeutics is the induction of TLSs by therapeutic intervention. Deciphering the aspects of the TME that do or do not allow TLS formation may represent a new opportunity to identify TLS inducers with sufficient safety and efficacy for direct clinical applications in cancer treatment. TLS formation can be induced by ICB with or without conventional radio-chemotherapy or by tumor-targeted delivery of chemokines and cytokines involved in TLS formation, such as LTα, LIGHT, and CXCL13. TLS formation can also be induced by therapeutic vaccination of tumor patients. Importantly, further investigations are needed to decipher the cellular and molecular mechanisms underlying these strategies, which may help elucidate why some patients respond to therapies and others do not. This can be achieved through the use of spheroid or organoid study models, which may better recapitulate the cellular complexity of the human TME than mouse models, leading to better prediction of patient responses to treatments. As a completely new area of research for which little data currently exist, the study of TLSs may bring forth a new era of immunotherapy in the not-too-distant future.

## Author contributions

QZ prepared the manuscript and revised the paper. SW provided advanced guidance for its preparation. All authors contributed to the article and approved the submitted version.
